# High triiodothyronine levels induce myocardial hypertrophy via BAFF overexpression

**DOI:** 10.1111/jcmm.17470

**Published:** 2022-07-08

**Authors:** Guo‐Qing Li, Xiao‐Mei Liu, Bing‐Li Liu, Yi Zhong, Qing‐Wei Gu, Jing‐Jing Miao, Jie Wang, Shu Liu, Xiao‐Ming Mao

**Affiliations:** ^1^ Department of Endocrinology, Nanjing First Hospital Nanjing Medical University Nanjing China

**Keywords:** B‐cell activating factor, Graves' disease, inflammatory response, myocardial hypertrophy, triiodothyronine

## Abstract

Activated B cells contribute to heart diseases, and inhibition of B‐cell activating factor (BAFF) expression is an effective therapeutic target for heart diseases. Whether activated B cells participate in the development and progression of hyperthyroid heart disease, and what induces B cells activation in hyperthyroidism are unknown. The present study aimed to determine the roles of BAFF overexpression induced by high concentrations of triiodothyronine (T3) in the pathogenesis of hyperthyroid heart disease. Female C57BL/6J mice were subcutaneously injected with T3 for 6 weeks, and BAFF expression was inhibited using shRNA. Protein and mRNA expression of BAFF in mouse heart tissues evaluated via immunohistochemistry, western blotting and polymerase chain reaction (PCR). Proportions of B cells in mouse cardiac tissue lymphocytes were quantified via flow cytometry. Morphology and left ventricle function were assessed using pathological sections and echocardiography, respectively. Here, we demonstrate that compared with the control group, the proportion of myocardial B cells was larger in the T3 group; immunohistochemistry, western blotting and PCR analyses revealed increased protein and mRNA expression levels of TNF‐α and BAFF in heart tissues of the T3 group. Compared with the normal controls group, in the T3 group, the diameter of myocardial cells and some echocardiographic values significantly increased and hypertrophy and structural disorder were noticeable. Our results revealed that elevated levels of circulating T3 can promote the expression of BAFF in myocardial cells and can lead to B‐cell activation, an elevated inflammatory response and ventricular remodelling.

## 1 INTRODUCTION

High thyroid hormones (THs; mainly T3 and T4) levels play crucial roles in hyperthyroid heart disease; even minor changes in circulating TH concentrations can adversely affect the cardiovascular system.^1^ Overt hyperthyroidism increases the risk of atrial fibrillation and is associated with hyperdynamic states.^2^ Cardiac output increases substantially due to reduced systemic vascular resistance and increased preload, contractility and heart rates.^2^ THs affect cardiac status via direct genomic actions on cardiomyocytes by binding to nuclear receptors, which leads to the regulation of the expression of target genes; via extranuclear, non‐genomic actions on the ion channels in cardiomyocyte membranes; and by altering peripheral circulation, which determines cardiovascular haemodynamics, cardiac filling and systolic contractility.^1^ However, these actions of THs cannot fully explain the pathogenesis of hyperthyroid heart disease.

Long term hyperthyroidism manifests mainly due to onset of Graves' disease (GD). Innate and adaptive immune system disorders play key roles in cardiovascular disease (CVD),^3^, ^4^ and the activation status of B cells is a key factor in determining CVD risk.^5^ Mature B lymphocytes orchestrate various adaptive immune responses relevant to human diseases^6^; B‐cell activation triggers monocyte mobilization and impairs heart function and vascular injury.^7^, ^8^ High levels of circulating T3 can affect the activation and differentiation of B cells, stimulate plasma cytogenesis (favouring bone marrow) and prolong plasma cell survival.^9^ However, the mechanisms underlying the activation and differentiation of B cells by THs, and whether they participate in hyperthyroid heart disease, are unknown.

B‐cell activating factor (BAFF) is a type of tumour necrosis factor (TNF) that plays key roles in the survival, differentiation and maturation of B cells.^10^ The expression of BAFF is abnormal during the occurrence and development of various autoimmune diseases.^11^ Elevated serum levels of BAFF in patients with rheumatoid arthritis are associated with autoantibody levels and synovitis.^12^ BAFF level is also elevated in patients with GD.^13^ Although the mechanism of elevated BAFF levels in these autoimmune diseases is unclear, anti‐BAFF antibodies might be a useful target for treating autoimmune and myocardial diseases. Inhibition of BAFF is effective against systemic lupus erythematosus and rheumatoid arthritis.^14^, ^15^ B‐cell depletion reduces the development of atherosclerosis,^16^ as well as areas of cardiac infarction and fibrosis, and improves ventricular function in BAFF receptor‐deficient mice and mouse models with B lymphocytes depleted by anti‐BAFF antibodies.^17^

THs play key roles in the energy balance of the heart, and high THs levels can lead to elevated metabolic states. However, the role of elevation in TH levels in the immune system is unknown. We explored the roles of THs in hyperthyroid heart disease in mouse models by subcutaneously injecting mice with a high concentration of T3. We then determined proportions of B cells, as well as BAFF and TNF‐α expression, in cardiac tissues of these mice and evaluated their cardiac functions.

## 2 MATERIAL AND METHODS

### 2.1 Animals

Six‐week‐old C57BL/6J mice weighing 17–19 g (Experiment Animal Center of Nanjing First Hospital, Nanjing Medical University) were maintained under specific pathogen‐free conditions. They were bred and housed in individual cages with free access to standard laboratory water and chow. All animal procedures complied with the guide for the Care and Use of Laboratory Animals published by the animal care committee of Nanjing First Hospital, Nanjing Medical University and the National Institute of Health and were reviewed and approved by the Ethics Committee of Laboratory Animals of Nanjing First Hospital, the First Affiliated Hospital of Nanjing Medical University.

### 2.2 Experimental design and specimen preparation

The mice were randomly assigned to control, T3, negative control shRNA+T3 (NCs + T3), and BAFF shRNA+T3 (Bs + T3) groups, each group was 8 mice. The tail veins of NCs + T3 and Bs + T3 groups were injected intravenously with NCs and Bs, respectively (Genechem Co., Ltd.). Four weeks later, the T3, NCs + T3 and Bs + T3 groups were injected subcutaneously with T3 (5 μg/10 g) (Meilun Biotech Co., Ltd.) every day for 6 weeks, and the control group was injected subcutaneously with the same volume of saline. Mice were tagged by toe clipping and weighed daily before and after administration. Daily amounts of ordinary feed (Jiangsu Syony Pharmaceutical Biological Engineering Co. LTD., Nanjing, China. Iodine ≤0.6 mg/kg) and water were recorded for observing the symptoms of hyperthyroidism. The mice were killed under chloral hydrate anaesthesia, 1 day after the end of the experiments, and blood was extracted from the atrium. The hearts were weighed, cut and rinsed with 0.9% sodium chloride, and then, the atrium and ventricles were separated. Ventricles were cut into three pieces for pathological sectioning, immunohistochemical staining, western blotting and reverse transcription polymerase chain reaction (RT‐PCR).

### 2.3 Lentiviral construction

GV248 was selected as the BAFF inhibitory lentiviral vector. The sequence of the vector component was hU6‐MCS‐Ubiquitin‐EGFP‐IRES‐puromycin, and the RNA interference target sequence was designed based on the BAFF gene sequence, CGGGAGAATGCACAGATTT, and the control sequence was TTCTCCGAACGTGTCACGT.

Then double‐stranded DNA was synthesized, digested and inserted into GV248 vector by T4 DNA ligase, transformed into receptor cells and plasmid was extracted. Viruses were packaged, amplified in 293 T cells and purified. Viruses in 293 T cells were assayed using Adeno‐X Rapid Titre kits (Takara Bio Inc., Kusatsu, Japan), as described by the manufacturer.

### 2.4 Echocardiographic evaluation

A single investigator who was blinded to the experimental groups conducted cardiac measurements using M‐mode echocardiography with a 17.5 MHz linear array, Vevo2100™ High Resolution Imaging System (Fujifilm VisualSonics, Fujifilm Holdings Corporation) at the end of the study. The following structural parameters were evaluated: left ventricular internal dimension in diastole (LVIDd), left ventricular internal dimension in systole (LVIDs), interventricular septal thickness in systole (IVSs) and in diastole (IVSd), and LV posterior wall thickness in systole (LVPWs) and diastole (LVPWd). Left ventricular mass was calculated as [(LVIDd + LVPWd + IVSd)3 − (LVIDd) 3 × 1.04 × 0.8 + 0.6] and LV function was assessed based on fractional shortening (FS) and ejection fraction (EF).

### 2.5 Measurement of serum levels of T3 and T4

Blood was collected from mouse atria and centrifuged at 250 *× g* for 5 min. Serum was stored at −80°C. Serum concentrations of T3 and T4 in serum were determined using radioimmunoassays.

### 2.6 Measurement of heart index

Mice hearts were weighed to obtain heart indexes (ratio of heart weight to body weight [mg/g]).

### 2.7 Determination of myocardial cell diameter

Morphological changes were assessed in dewaxed and haematoxylin‐eosin (HE)‐stained paraffin sections of myocardial cells using light microscopy at 200× magnification. The shortest transverse diameters of myocardial cells with clear boundaries were measured at the nuclear level. Five fields were selected from each section, and 10 cells were examined per field. The mean value was taken as the transverse diameter of the myocardial cells.

### 2.8 Immunohistochemical staining heart tissue

Heart tissues preserved in 10% formalin were dehydrated, embedded in paraffin, dewaxed and incubated at room temperature with 3% H_2_O_2_; then, antigens were retrieved using 0.01 mol/L citric acid solution at high temperature. The sections were immunohistochemically stained using anti‐BAFF antibody (Abcam plc., Cambridge, UK) and visualized with DAB‐H_2_O_2_, then dehydrated, stained with haematoxylin and sealed with neutral gum for light microscopy. The cytoplasm of BAFF‐positive cells was visualized as a brownish yellow stain using a pathological image analysis system. Five visual fields were randomly selected from each section and assessed using a light microscope at 100× magnification. Integral absorbance was calculated as levels of positive BAFF expression.

### 2.9 Western blotting

The ventricle tissues were homogenized, and total proteins obtained using Tissue and Cell Total Protein Extraction Kits (Jiangsu KeyGEN BioTECH Corp., Ltd) were used to analyse BAFF and TNF‐α expression. Total protein (20 μg) was resolved via 15% SDS‐PAGE (Beyotime Biotech Co., Ltd., Shanghai, China) and then transferred to polyvinylidene fluoride membranes. Nonspecific protein binding on the membranes was blocked with 5% non‐fat milk for 2 h, followed by incubation with anti‐BAFF (Abcam plc.), anti‐TNF‐α (Proteintech Group, Inc.) and anti‐β‐actin (Cell Signalling Technology Inc.) antibodies overnight at 4°C. The anti‐β‐actin was selected as reference according previous study^18^ and due to the molecular weight of BAFF being close to that of anti‐GAPDH. After three washes with TBST buffer, the membranes were incubated with horseradish peroxidase‐conjugated secondary antibody at room temperature for 2 h. Proteins were detected using an enhanced chemiluminescence system and quantified using image J software.

### 2.10 Quantitative real‐time PCR

Ventricles were ground in liquid nitrogen, and total RNA was extracted using TRIzol (KeyGEN BioTECH Corp., Ltd., Nanjing, China). The RNA precipitate was diluted 100‐fold with deionized water, quantified via spectrophotometry and then reverse transcribed into cDNA using PrimeScript™ RT Reagent Kits with gDNA Eraser (Takara Bio Inc.), as described by the manufacturer, for two‐step RT‐PCR. Reverse transcriptase was omitted from the negative control to determine whether genomic DNA remained in the cDNA. PCR amplification proceeded in a mixture containing 10 μl of SYBR® Premix Ex Taq (2×), 2 μl of cDNA, 0.8 μl of either upstream or downstream primers, 0.4 μl of ROX Reference Dye II (50×) and 6 μl of double steam‐sterilized water. The cycle parameters were 95°C for 30 s, followed by 40 cycles of 95°C for 5 s and 60°C for 34 s. In order to analyse relative gene expression with real‐time PCR, we compared three reference genes: β‐actin, glyceraldehyde 3‐phosphate dehydrogenase (GAPDH) and 18 s. The intra‐ and inter‐assay variation was tested. The real‐time PCR efficiency of β‐actin and GAPDH was similar, and expression of the two reference genes was constant. We selected β‐actin as reference gene and expression of the target gene was normalized to β‐actin as previous studies.^19^ The (5′→3′) forward and reverse primers were as follows: mouse BAFF, CCACCGTGCCTCTGTTTTTG and CTTCTGCGGAGTGATGGGAT; mouse TNF‐α, GATGGGGGGCTTCCAGAACT and TGGGGACCGATCACCCCGAA; mouse β‐actin, AGAGGGAAATCGTGCGTGAC and CAATAGTGATGACCTGGCCGT.

### 2.11 Cell separation and flow cytometry

Tissues were cut into fragments of 0.5–1‐mm^3^ using ophthalmic scissors and then transferred to 15 ml centrifuge tubes with an appropriate amount of D‐Hanks buffer. The cell suspension was passed through a 200‐mesh screen; then, the filtrate was centrifuged at 250 *× g* for 10 min and the supernatant was discarded. Pelleted cells were resuspended in diluent, layered on top layer of lymphocyte separation solution and centrifuged at 450 *× g* for 20 min. The second layer of flocculent lymphocytes was gently withdrawn and washed with 5 ml of cleaning solution. We detected B220‐positive B cells by staining 50 μl lymphocytes with APC‐conjugated anti‐B220 antibodies (Becton Dickinson and Co.). Cells were sorted using a BD FACSCanto™‐II flow cytometer and analysed using FlowJo software. Dead cells were excluded based on scatter profiles and 7‐aminoactinomycin D staining during flow cytometry.

### 2.12 Statistical analysis

Data were statistically analysed using SPSS 23.0 software (IBM Corp.). Data are expressed as mean ± SD. Comparisons between groups were assessed using independent sample *t*‐tests, and values with *p* < 0.05 were considered statistically significant.

## 3 RESULTS

### 3.1 Changes in body weight and serum T3 and T4 levels after T3 administration

Serum T3 levels were significantly higher in mice administered T3 for 6 weeks than those in control mice (*p* < 0.001). Serum T4 was undetectable in mice administered with T3. Mice in the T3 group weighed slightly less than those in the control group after 6 weeks of T3 administration, but the difference did not reach significance. The average diet and water intake was significantly greater in the T3 group, compared with the control group (Table 1).

**TABLE 1:** Changes in body weight and serum T3 and T4 levels after T3 administration

	T3 (nm/L)	T4 (nm/L)	Weight (g) 0 day	Weight (g) 42 days	Diet amount (g)	Water intake (ml)
Control	0.74 ± 0.16	**1**4.59 ± 0.86	17.79 ± 0.64	20.89 ± 1.69	130.74 ± 5.54	141.70 ± 6.34
T3	2.80 ± 1.19***	Not detectable	17.96 ± 0.55	20.24 ± 1.28	200.93 ± 7.15***	272.13 ± 10.15***

*Note:* The serum levels of T3, T4 and weight of mice in different group were analysed by Student's *t*‐test. The values are shown as Mean ± SD.

*** *p* < 0.001, T3 group compared to control (*n* = 8 mice per group).

### 3.2 Elevated levels of T3 affect myocardial cell diameter and myocardial hypertrophy (MH)

**FIGURE 1:**
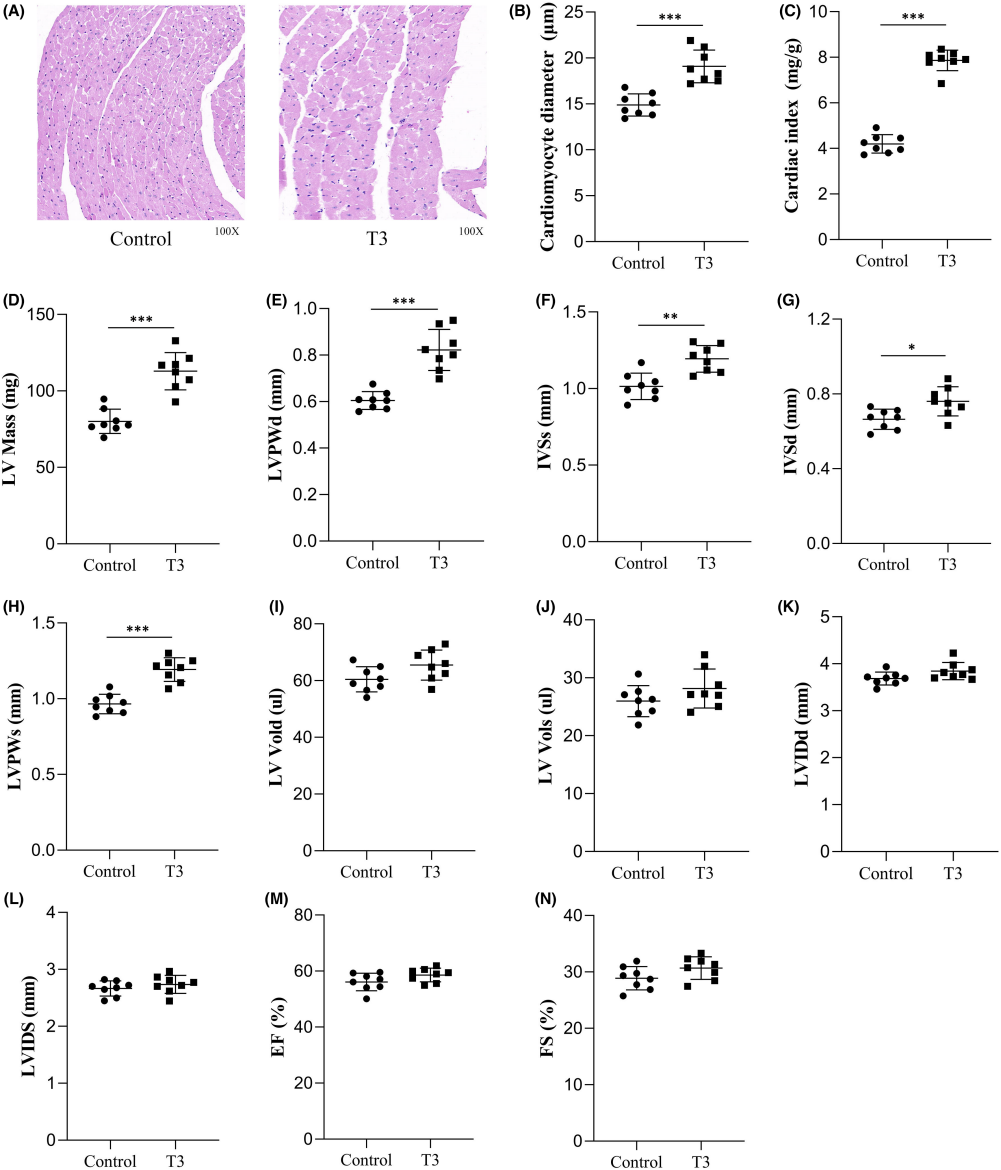
Elevated levels of T3 affect myocardial cell diameter and myocardial hypertrophy (MH). The heart sections were stained with HE and examined by light microscopy at 200× magnification. (A) Representative histological photomicrographs of the heart in control group and T3 group. (B) The cardiomyocyte diameter, bar graphs represent mean ± SD, ***p < 0.001, T3 group compared with control group (*n* = 8 mice per group). (C) The cardiac Index, bar graphs represent Mean ± SD, ***p < 0.001, T3 group compared with control group (*n* = 8 mice per group). (D–N) The statistical analysis of LV mass, LVPWd, IVSs, LVSd, LVPWs, LV Vold, LV Vols, LVIDd, LVIDs, EF and FS in the heart of mice in control and T3 group (*n* = 8 mice per group), bar graphs represent Mean ± SD, *p < 0.05, **p < 0.01, ***p < 0.001

Myocardial tissue sections stained with HE revealed myocardial cells with a significantly larger transverse diameter in the T3 group, compared with the control group (*p* < 0.001; Figure 1A,B), and the heart index was significantly higher in the T3 group (*p <* 0.001; Figure 1C).

**FIGURE 2:**
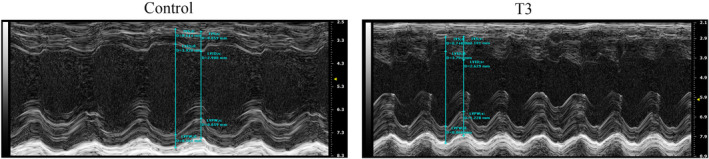
LV echocardiographic representative images in control and T3 groups

We explored whether high T3 levels affected LV dimension and cardiac function in mice by measuring cardiac parameters obtained by M‐mode and Doppler echocardiography. Figure 1D–G shows that LV mass, LVPWd, IVSs, LVSd and LVPWs in the T3 group, compared with the control group (*p* < 0.001, *p* < 0.01 and *p* < 0.05, respectively), whereas values for, LV Vold, LV Vols, LVIDd, LVIDs, EF and FS did not significantly differ between the two groups (*p* > 0.05 for all; Figure 1H–N). The LV echocardiographic images in Control and T3 groups were showed in Figure 2.

### 3.3 Frequency of B cells in myocardial tissues

**FIGURE 3:**
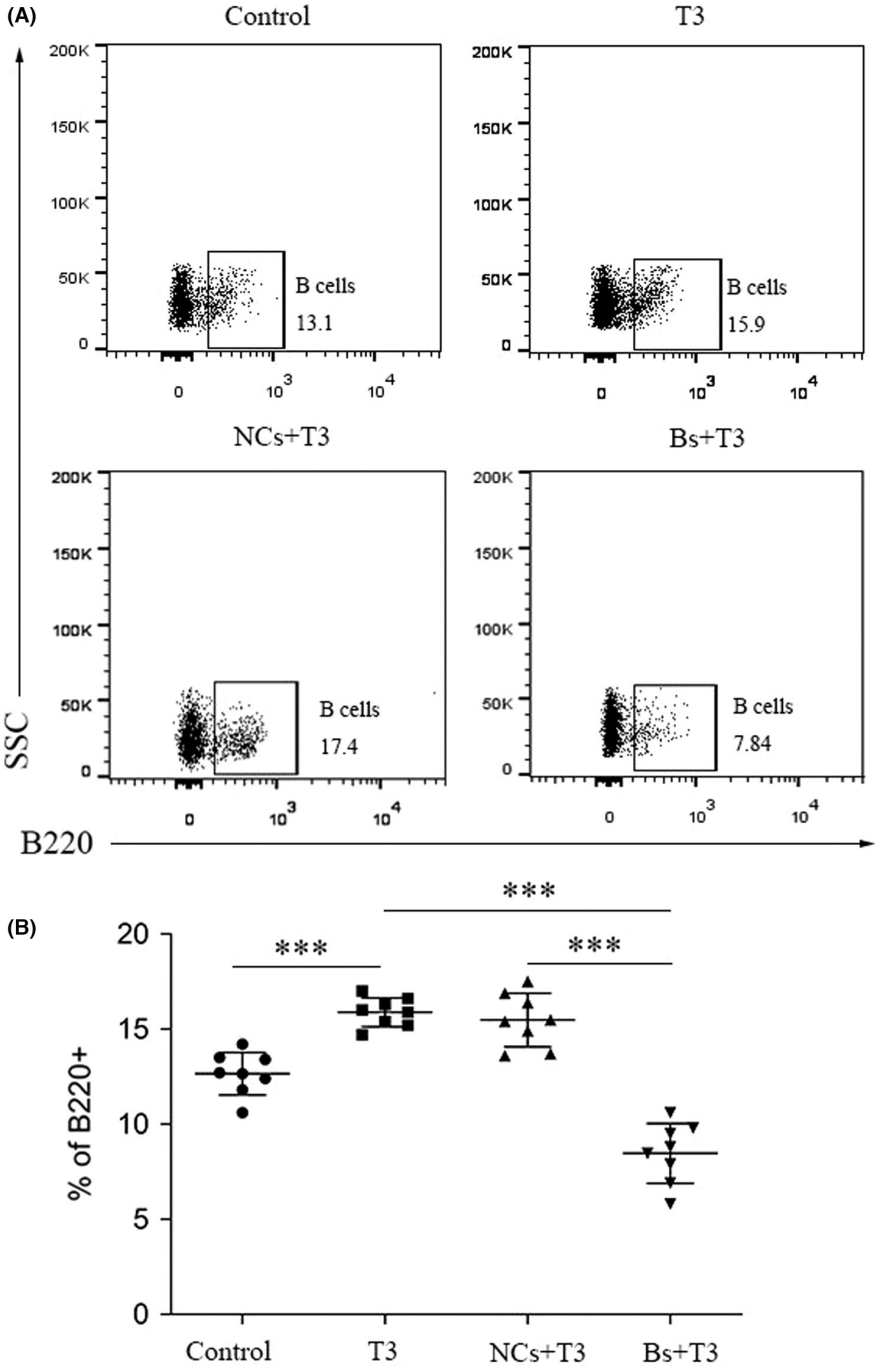
Frequency of B cells in myocardial tissues. The mononuclear cells in heart tissues were isolated and submitted to flow cytometry using anti‐mouse B220‐APC. (A) Representative the frequency of B220+ B cells of the four groups in heart tissues. (B) Bar graphs represent Mean ± SD, ***p < 0.001, T3 group compared with control group, ***p < 0.001, Bs + T3 group compared with NCs + T3 group (*n* = 8 mice per group)

The frequency of B cells in myocardial tissues was determined via flow cytometry. The proportion of B cells in the myocardium was significantly higher in the T3 group, compared with the control group (*p* < 0.001; Figure 3A,B). The proportion of B cells was significantly reduced in the Bs + T3 group, compared with the NCs + T3 group and T3 group (*p* < 0.001; Figure 3A,B).

### 3.4 Overexpression of BAFF and TNF‐α was induced by T3

**FIGURE 4:**
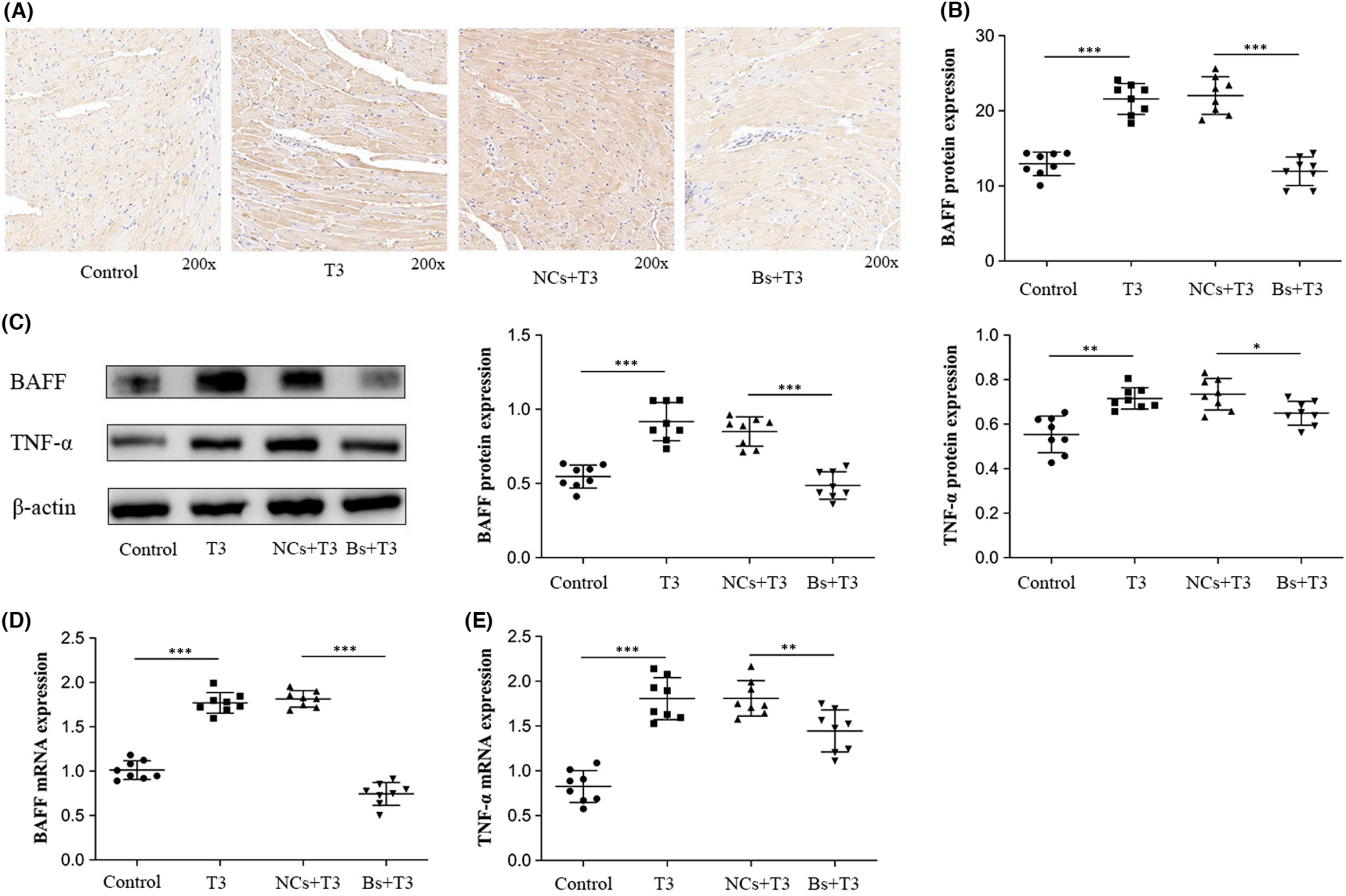
Overexpression of BAFF and TNF‐α was induced by T3. (A and B) Representative immunohistochemical stain of BAFF protein of the heart tissues in control group, T3 group, NCs + T3 group and Bs + T3 group. Bar graphs represent Mean ± SD, ***p < 0.001, T3 group compared with control group and Bs + T3 group compared with NCs + T3 group (*n* = 8 mice per group). (C) For western blot protein analysis of BAFF and TNF‐α in mice heart tissues, β‐Actin was used as house‐keeping protein. Bar graphs represent Mean ± SD, **p < 0.01, *p < 0.05, ***p < 0.001, respectively. T3 group compared with control group (*n* = 8 mice per group) and Bs + T3 group compared with NCs + T3 group. (D) The BAFF mRNA expression in the heart tissues, bar graphs represent Mean ± SD, ***p < 0.001, T3 group compared with control group and Bs + T3 group compared to NCs + T3 group (*n* = 8 mice per group). (E) The TNF‐α mRNA expression in the heart tissues, bar graphs represent Mean ± SD, ***p < 0.001 and **p < 0.01, T3 group compared with control group and Bs + T3 group compared to NCs + T3 group (*n* = 8 mice per group)

Immunohistochemical staining revealed that the expression of BAFF in the heart of T3 group mice significantly increased, compared with the control mice (*p* < 0.001; Figure 4A,B). The level of BAFF expression was significantly reduced in hearts isolated from the Bs + T3 group, compared with the NCs + T3 group (*p* < 0.001; Figure 4A,B). The protein and mRNA expression levels of BAFF (Figure 4C,D) and TNF‐α (Figure 4C,E) were significantly higher in the heart tissues of the T3 group mice, compared with the control group mice (*p* < 0.001 and *p* < 0.01, respectively). Protein and mRNA expression levels of BAFF (Figure 4C,D) and TNF‐α (Figure 4C,E) in heart tissues were significantly lower in the Bs + T3 group, compared with the NCs + T3 group (*p* < 0.001, *p* < 0.01 and *p* < 0.05, respectively).

### 3.5 Inhibition of BAFF expression improved MH

**FIGURE 5:**
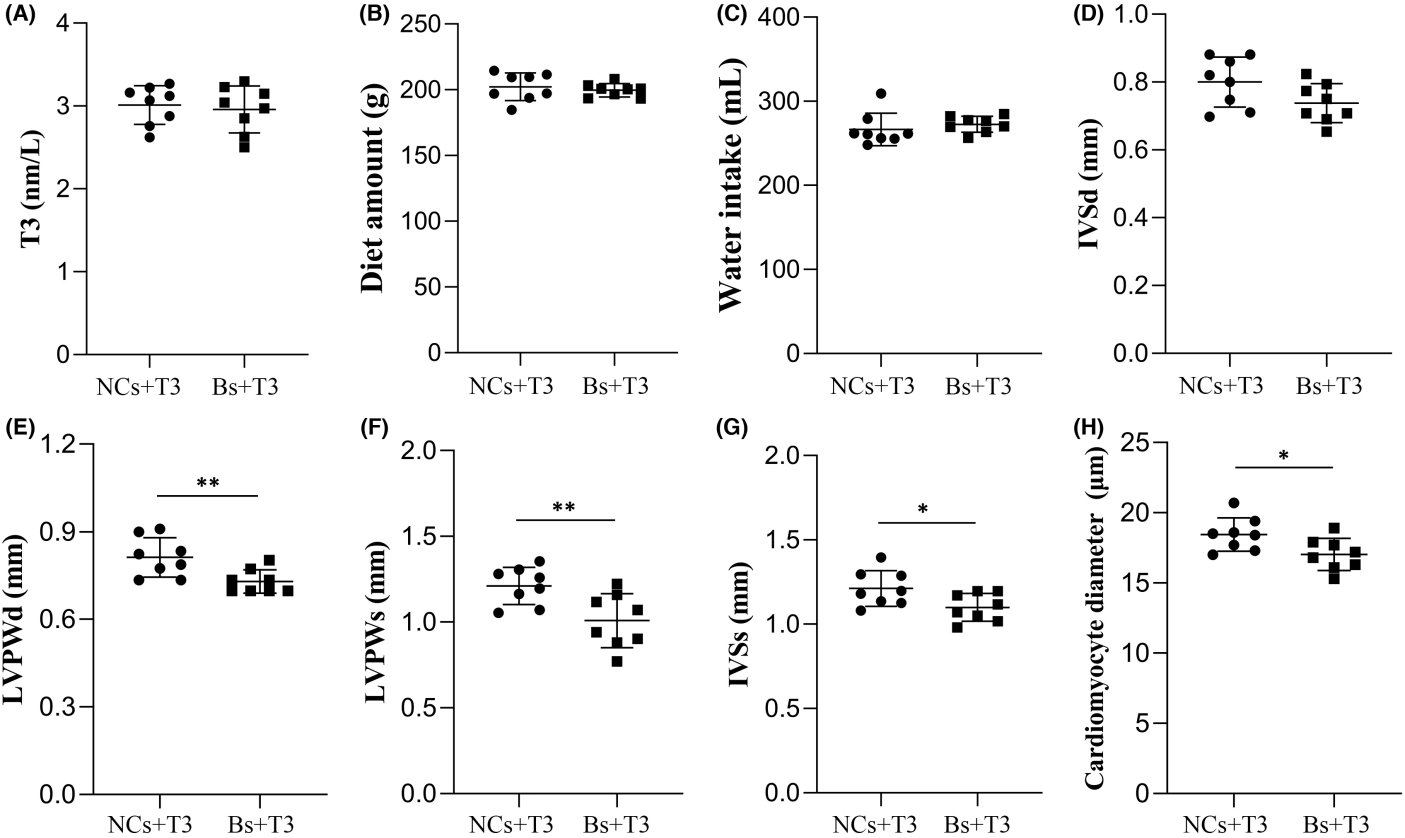
Inhibition of BAFF expression improved MH. (A) The serum levels of T3 in NCs + T3 and Bs + T3 group, bar graphs represent Mean ± SD, *p* > 0.05. (B and C) The diet amount (B) and water intake (C) of each mice in NCs + T3 and Bs + T3 group, bar graphs represent Mean ± SD, *p* > 0.05. (D) The cardiomyocyte diameter of mice heart tissues in NCs + T3 and Bs + T3 group, bar graphs represent Mean ± SD, *p < 0.05. (E–H) The statistical analysis of IVSd (E), IVSs (F), LVPWd (G) and LVPWs (H) of mice heart of in NCs + T3 and Bs + T3 group, bar graphs represent Mean ± SD, *p < 0.05, **p < 0.01 (*n* = 8 mice per group)

**FIGURE 6:**
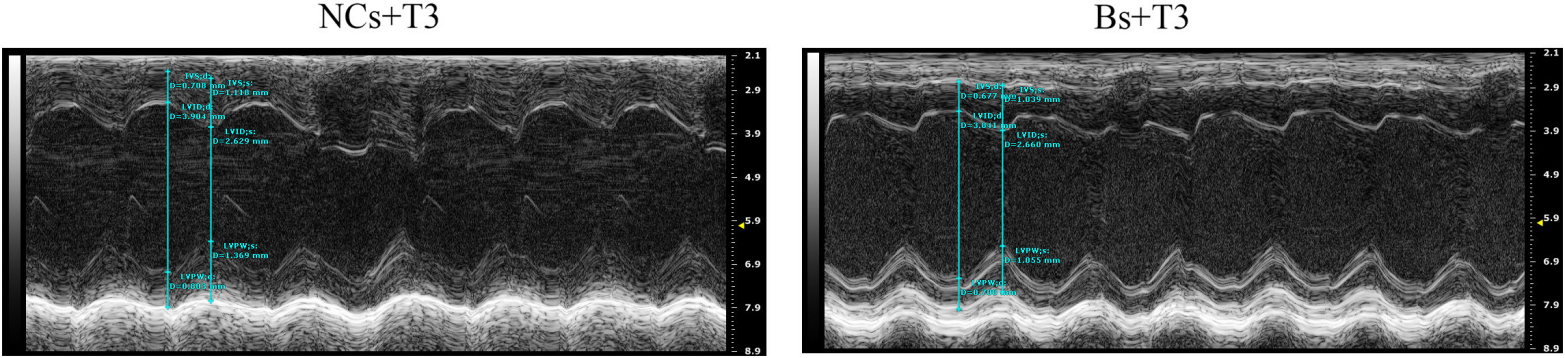
LV echocardiographic representative images in NCs + T3 and Bs + T3 groups

To confirm that high T3 levels impair cardiomyocytes and cause MH through BAFF overexpression, we used a lentivirus to interfere with BAFF expression. The serum T3 levels, food, water intake and IVSd did not significantly differ between these groups (*p* > 0.05 for all; Figure 5A–D). The values of LVPWd, LVPWs, LVSs and diameter of myocardial cells were significantly lower in the Bs + T3 group, compared with the NCs + T3 group (*p* < 0.01 and *p* < 0.01, respectively; Figure 5E–H). The LV echocardiographic images in Bs + T3 and NCs + T3 groups were showed in Figure 6.

## 4 DISCUSSION

We revealed that elevated levels of T3 promoted BAFF expression in mouse heart tissues, led to the activation of B cells and promoted a nonspecific inflammatory response. Inhibiting BAFF expression attenuated the damage to the myocardium induced by high T3 levels. Overexpression of BAFF and activation of B cells by high THs levels might play important roles in the development and progression of hyperthyroid heart disease.

Mice administered T3 (5 μg/10 g of body weight per day) for 6 weeks developed a 3–4‐fold increase in serum T3 levels, which was consistent with previous findings.^9^ We applied a relatively high dose of T3 to the mice for a longer period to induce pathological hypertrophy and impaired heart function through B‐cell activation. However, we did not identify any changes in EF and FS and exclude the possibility of physiological hypertrophy occurring in the T3 group mice. It is difficult to differentiate between short‐term physiological and pathological hypertrophy in experimental animals administered with T3.^20^ Although hyperthyroid heart disease is a chronic complication of hyperthyroidism, MH is an independent risk factor for cardiovascular accidents and the development of heart failure. The incidence of cardiovascular accidents is 2–4‐fold higher in patients with MH, compared to those without MH.^21^, ^22^ Cardiac pathologies range from physiological cardiac hypertrophy with enhanced function to cardiac dilation and heart failure.^23^

We also found that high T3 levels induced BAFF overexpression and increased the proportion of B cells in mouse heart tissues. Neutrophils, monocytes, dendritic cells, and lymphatic stromal cells^24^ and some tissue epithelial cells^25^, ^26^ express BAFF. However, the exact sources of BAFF in heart tissues require further investigation. BAFF stimulates B‐cell maturation and proliferation and prolongs B cells survival by binding to its receptors on the surfaces of B cells.^10^, ^27^ Decreased BAFF expression levels lead to a deficiency of B cells, and the addition of BAFF to the circulatory system promotes B‐cell proliferation and serum antibody generation.^28^ Although serum BAFF levels are higher in patients with GD,^13^ little is known about BAFF expression in heart tissues, in addition to what induces its overexpression and its roles in the development of hyperthyroid heart disease.

The proportion of B cells decreased in heart tissues of T3 mice when shRNA interfered with BAFF expression. Correspondingly, the thickness of the LV posterior wall and systolic ventricular septum decreased, indicating that BAFF and abnormal B‐cell proliferation play crucial roles in the development of hyperthyroid heart disease.

The overexpression of BAFF can induce a tissue inflammatory reaction that plays a key role in the pathogenesis of various autoimmune diseases.^29^ A chronic nonspecific inflammatory response also plays a key role in the development of hyperthyroid heart disease.^30^ B cells can secrete inflammatory cytokines such as IL‐6 and TNF‐α,^31^, ^32^ and IL‐1β and TNF‐α directly promote cardiomyocyte hypertrophy and apoptosis.^33^ B lymphocytes cause myocardial dysfunction in dilated cardiomyopathy by secreting TNF‐α.^34^ Rituximab can improve cardiac remodelling and dysfunction caused by stress by reducing the ability of B lymphocytes to secrete proinflammatory factors.^35^ In BAFF receptor‐deficient mice and model mice depleted of B lymphocytes using anti‐BAFF antibodies, areas of cardiac infarction and fibrosis are reduced; ventricular function is improved; and IL‐1β, TNF‐α and IL‐18 expression is downregulated.^36^ Levels of inflammatory cytokines in activated B cells and in the circulatory system are significantly increased during acute decompensated heart failure.^30^ Furthermore, the activation of various B‐cell subsets in the adventitia of the aorta is significantly associated with atherosclerosis, and the depletion of B2 cell subsets significantly reduces the occurrence of atherosclerosis.^37^ We noted increased TNF‐α level in cardiomyocytes of T3 mice, indicating an elevated cardiac inflammatory response. The number of B lymphocytes decreased when shRNA was applied to interfere with BAFF expression; additionally, TNF‐α level decreased in mouse cardiomyocytes. Our findings suggest that TNF‐α secretion by B cells participates in cardiac remodelling.

We must acknowledge that there are some limitations to our study. First, the changes of inflammatory cytokines induced by T3 in the circulatory system were not available in our study, which may partially reflect the effects of inflammatory cytokines on heart. Second, we did not identify any changes in EF and FS, which may be due to the relative short period of hyperthyroidism in the T3 treated mice. Hyperthyroid heart disease is a chronic complication of hyperthyroidism and markedly decreased in EF and FS are indexes of heart failure.^38^ We believe that the decline of EF and FS will be observed in the mice with enough longer period of hyperthyroidism.

In summary, we have demonstrated for the first time that high T3 levels can induce BAFF overexpression and activate B cells in heart tissues of mice, which are followed by the induction of an inflammatory response and the restructuring of the left ventricular structure. Silencing BAFF expression using shRNA before injecting T3 can prevent MH in mouse models with T3‐induced hyperthyroidism. The current study elucidated a novel mechanism underlying BAFF‐mediated B‐cell activation in the development of hyperthyroid heart disease.

## AUTHOR CONTRIBUTIONS

**Guo‐Qing Li:** Conceptualization (equal); investigation (equal); methodology (equal); writing – original draft (equal); writing – review and editing (equal). **Xiao‐Mei Liu:** Data curation (lead); formal analysis (equal); funding acquisition (equal); investigation (equal); methodology (equal); supervision (equal); writing – original draft (equal). **Bing‐Li Liu:** Formal analysis (equal); funding acquisition (equal); investigation (equal); methodology (equal); supervision (equal); writing – original draft (equal). **Yi Zhong:** Investigation (equal); methodology (equal); project administration (equal); writing – review and editing (equal). **Qing‐Wei Gu:** Formal analysis (equal); investigation (equal); methodology (equal). **Jing‐Jing Miao:** Investigation (equal). **Jie Wang:** Methodology (equal). **Shu Liu:** Data curation (equal). **Xiao‐ming Mao:** Conceptualization (lead); data curation (lead); funding acquisition (equal); supervision (lead); writing – review and editing (lead).

## CONFLICT OF INTEREST

The authors have stated explicitly that there is no conflict of interest in connection with this article.

## Data Availability

The data that support the findings of this study are available from the corresponding author upon reasonable request.
